# Sugarcane responses to two strains of *Xanthomonas albilineans* differing in pathogenicity through a differential modulation of salicylic acid and reactive oxygen species

**DOI:** 10.3389/fpls.2022.1087525

**Published:** 2022-12-15

**Authors:** Jian-Ying Zhao, Juan Chen, Yang Shi, Hua-Ying Fu, Mei-Ting Huang, Philippe C. Rott, San-Ji Gao

**Affiliations:** ^1^ National Engineering Research Center for Sugarcane, Fujian Agriculture and Forestry University, Fuzhou, Fujian, China; ^2^ CIRAD, UMR PHIM, Montpellier, France, and PHIM Plant Health Institute, Univ Montpellier, CIRAD, INRAE, Institut Agro, IRD, Montpellier, France

**Keywords:** xanthomonas albilineans, genetic divergence, pathogenicity, Reactive oxygen species (ROS) homeostasis, Salicylic acid (SA) signaling pathway, defense response, sugarcane

## Abstract

Leaf scald caused by *Xanthomonas albilineans* is one of the major bacterial diseases of sugarcane that threaten the sugar industry worldwide. Pathogenic divergence among strains of *X. albilineans* and interactions with the sugarcane host remain largely unexplored. In this study, 40 strains of *X. albilineans* from China were distributed into three distinct evolutionary groups based on multilocus sequence analysis and simple sequence repeats loci markers. In pathogenicity assays, the 40 strains of *X. albilineans* from China were divided into three pathogenicity groups (low, medium, and high). Twenty-four hours post inoculation (hpi) of leaf scald susceptible variety GT58, leaf populations of *X. albilineans* strain XaCN51 (high pathogenicity group) determined by qPCR were 3-fold higher than those of strain XaCN24 (low pathogenicity group). Inoculated sugarcane plants modulated the reactive oxygen species (ROS) homoeostasis by enhancing respiratory burst oxidase homolog (ScRBOH) expression and superoxide dismutase (SOD) activity and by decreasing catalase (CAT) activity, especially after infection by *X. albilineans* XaCN51. Furthermore, at 24 hpi, plants infected with XaCN51 maintained a lower content of endogenous salicylic acid (SA) and a lower expression level of SA-mediated genes (*ScNPR3*, *ScTGA4*, *ScPR1*, and *ScPR5*) as compared to plants infected with XaCN24. Altogether, these data revealed that the ROS production-scavenging system and activation of the SA pathway were involved in the sugarcane defense response to an attack by *X. albilineans*.

## 1 Introduction

The genus *Xanthomonas* of the *Xanthomonadaceae* family is formed by plant pathogenic bacteria infecting numerous crop plants, thus having a significant economic and agricultural impact (Jacques et al., 2016; Te Molder et al., 2021). A distinct characteristic of *Xanthomonas* species (Gram-negative bacteria) is the production of the extracellular polysaccharide xanthan. This compound contributes to pathogenicity of xanthomonads and the taxonomic name of the genus derived from the yellow (xanthós in Greek) appearance on culture media (Ryan et al., 2011; da Silva et al., 2017). Wide genome and pathogenic divergences are often observed at *Xanthomonas* species or pathovar level (Te Molder et al., 2021; Tambong et al., 2022).

*Xanthomonas albilineans* causes leaf scald, a vascular disease of sugarcane that occurs in almost all sugarcane-growing countries (Rott & Davis, 2000; Ntambo et al., 2019b). White-yellow ‘pencil’ lines running along the main leaf veins are characteristic of leaf scald, and this symptom gave the name to the pathogen’s species name. Other disease symptoms include leaf chlorosis and leaf necrosis that result in stalk or entire plant death in susceptible sugarcane varieties (Rott & Davis, 2000; Lin et al., 2018). Leaf scald can cause severe yield losses and reduction of juice quality, and even the loss of entire sugarcane fields (Ricaud & Ryan, 1989; Rott & Davis, 2000; Ntambo et al., 2019b).

High immunological and genetic diversity exists among *X. albilineans* strains. Twenty-eight isolates from 11 different countries were divided into three serovars and six lysovars (Rott et al., 1986). Occurrence of three serovars within *X. albilineans* was confirmed with an additional set of 215 strains of the pathogen from 28 different geographical locations (Rott et al., 1994b). Thirty-eight strains of *X. albilineans* from different countries were also distributed into three main groups and eight subgroups based on DNA fingerprinting and serological reactions with monoclonal antibodies (Alvarez et al., 1996). Using a worldwide collection of isolates, 54 genetic haplotypes and eight pulsed-field gel electrophoresis (PFGE) groups (A-H) were identified within the leaf scald pathogen by genome mapping (Davis et al., 1997). Later on, three additional PFGE groups (I and J) were identified, and this genomic variation was confirmed by multilocus sequence analysis (MLSA) and genomics (Pieretti et al., 2012; Ntambo et al., 2019b; Zhang et al., 2020). Congruent genetic divergence within *X. albilineans* populations was also reported using fingerprinting methods such as random amplified fragment polymorphism (RAPD), rep-PCR, amplified fragment length polymorphism (AFLP), and simple sequence repeats (SSR) (Permaul et al., 1996; Lopes et al., 2001; Shaik et al., 2009; Tardiani et al., 2014).

Besides immunological and genetic divergences, variation in pathogenicity was also reported for the sugarcane leaf scald pathogen. For example, strains of *X. albilineans* belonging to different serovars varied in pathogenicity based on multiplication of the pathogen in sugarcane varieties (Mohamed et al., 1996). High variation in disease severity (DS) among strains of *X. albilineans* was found in Australia, Brazil, Guadeloupe, Mexico, and in the Guangxi province of China (Persley, 1973; Champoiseau et al., 2006b; Huerta-Lara et al., 2009; Tardiani et al., 2014; Wu et al., 2022). Notably, significant strain effects and variety × strain interactions were observed for strains originating from different countries (Mohamed et al., 1996). In contrast, no correlation was found between genetic variants or haplotypes and variation in pathogenicity of the leaf scald pathogen (Champoiseau et al., 2006b; Tardiani et al., 2014).

Pathogenicity factors of *X. albilineans* are involved in sugarcane colonization and disease progress including epiphytic survival, sensitivity to oxidative stress, plant cell adhesion, plant cell degradation by extracellular enzymes, bacterial motility, and biofilm development (Rott et al., 2013; Mensi et al., 2016; Mielnichuk et al., 2021). Albicidins produced by *X. albilineans* are a family of potent antibiotics and phytotoxins that inhibit DNA replication in bacteria and plastids (Birch and Patil, 1987b; Birch, 2001). *In planta*, albicidin production is closely associated with appearance of leaf pencil lines and chlorosis (Birch and Patil, 1987a). Mutagenesis of locus XALc_0557, coding for an OmpA protein of the *X. albilineans* cell membrane, strongly affects sugarcane stalk colonization by the pathogen (Rott et al., 2011a; Fleites et al., 2013). Other putative pathogenicity genes of *X. albilineans* include ABC transporter genes, a methyl-accepting chemotaxis protein gene, a gene conferring resistance to novobiocin, and an oxidoreductase gene (Rott et al., 2011b; Pieretti et al., 2012).

Plants are continuously challenged by diverse pathogens and have evolved a two-tiered immunity system, namely pattern-triggered immunity (PTI) and effector-triggered immunity (ETI) (Tabassum and Blilou, 2022; Ngou et al., 2022). At PTI stage, the pathogen recognized by the plant activates a complex network of signaling pathways such as reactive oxygen species (ROS), mitogen-activated protein kinase (MAPK), Ca^2+^ pathways, and hormone signaling that constitute the plant’s basal defense response (Tabassum and Blilou, 2022). Salicylic acid (SA) biosynthesis and SA-mediated signaling pathway play a key role in plants for establishment of resistance to many pathogens (Ding and Ding, 2020). Genes involved in metabolic pathways, biosynthesis of secondary metabolites, MAPK signaling, hormone signal transduction, and plant-defense related pathways contribute to the response of sugarcane after an attack by *X. albilineans* (Meng et al., 2020; Ali et al., 2021; Javed et al., 2022; Ntambo et al., 2019a). The molecular mechanisms triggered in sugarcane after infection by strains of *X. albilineans* differing in pathogenicity have, however, not been explored so far.

This first objective of this study was to perform molecular genotyping of 40 strains of *X. albilineans* from China using MLSA and SSR methods. The second objective was to characterize the pathogenicity of these strains by inoculation under greenhouse conditions of a sugarcane variety susceptible to leaf scald. The third objective was to investigate the molecular mechanisms triggered by one strain of *X. albilineans* with low pathogenicity and another strain of the pathogen with high pathogenicity. Explored mechanisms included ROS production and scavenging, as well as SA content and SA-mediated signaling pathway regulation.

## 2 Materials and methods

### 2.1 *X. albilineans* strains and DNA extraction

Thirty-nine strains of *X. albilineans* were isolated from 38 sugarcane leaf or stalk samples and one leaf sample of *Pennisetum purpureum* (**Table S1**). All samples were taken from plants exhibiting leaf scald symptoms and were collected during 2018-2019. The diseased sugarcane plants originated in six provinces (Fujian, Guangdong, Guangxi, Guizhou, Hainan, and Zhejiang) and the diseased pennisetum was collected in Fujian province. Isolation of *X. albilineans* was performed as described by Lin et al. (2018). Reference strain Xa-FJ1 from China was added to this collection of *X. albilineans* (Zhang et al., 2020). Total genomic DNA was extracted from the 40 strains using the Bacterial Genomic DNA extraction kit and following the manufacturers’ protocol (Tiangen Biotechnology Co. Ltd, Beijing, China). All DNA samples were adjusted to a concentration of 100 ng/μL with sterile water, and then stored at 80 °C until further use.

### 2.2 Amplification and sequencing of bacterial housekeeping genes

Four housekeeping genes were amplified from extracted total DNA and sequenced as described by (Ntambo et al. 2019b). These four genes included *rpoD* (encoding the β subunit of the bacterial RNA polymerase), *glnA* (encoding a citrate synthase), *gyrB* (encoding the β subunit of the DNA gyrase), and *atpD* (encoding the β subunit of ATP synthase) (**Table S2**). The 156 gene sequences (4 genes x 39 strains) of *X. albilineans* obtained in this study were deposited at the NCBI GenBank database under accession numbers MT776038-MT776077 (*gyrB*), MT776128-MT776139 and ON112140-ON112166 (*rpoD*), MT776144-MT776155 and ON112168-ON112194 (*atpD*), and MT776160-MT776171 and ON112107-ON112138 (*glnA*).

### 2.3 Multilocus sequence analysis

For each of the 39 *X. albilineans* strains, the sequences of the four housekeeping genes were concatenated, thus yielding sequences of 4,165 nucleotides (nt) in length. The corresponding sequences of strain Xa-FJ1 from China and 14 additional strains of *X. albilineans* from different countries were also downloaded from the GenBank database (**Table S1**). The 54 sequences were aligned with the ClustalW algorithm implemented in MEGA 11 and a phylogenetic tree was constructed using the neighbor-joining (NJ) method (Tamura et al., 2021). Bootstrap values were determined for 1,000 replications. Classification of PFGE groups of each clade was performed as reported by Pieretti et al. (2012).

### 2.4 SSR genotyping through sequencing

Fifteen SSR markers developed by Tardiani et al. (2014) were used to assess the diversity and population structure of the 40 *X. albilineans* strains from China. The PCR reaction was performed in a volume of 20 µL consisting of 1x HotStarTaq buffer, 2.0 mM Mg^2+^, 0.2 mM dNTP, 0.2 U HotStarTaq polymerase (Qiagen Inc, Frankfurt, Germany), 1.0 µM each of the forward and reverse primers, and 10-30 ng/µl of genomic DNA. The reaction conditions included an initial denaturation step at 95 °C for 2 min, followed by 11 cycles of 94 °C for 20 s and 65 °C for 40 s for primer annealing, and an extension step at 72 °C for 2 min; and then 24 cycles of 94 °C for 20 s and 59 °C for 30 s for primer annealing, and an extension step at 72 °C for 2 min; the final step was an extension period at 72 °C for 10 min. The PCR products were diluted 10 times and 1 μL of the diluted product was denatured at 95 °C for 5 min and then mixed with 0.5 μL fluorescein standard (GeneScan 500 LIZ™, ABI) and 8.5 μL formamide (Hi-Di). Lastly, capillary electrophoresis of the PCR products was conducted on an ABI3730XL DNA sequencer (Applied Biosystems, Foster City, CA, USA) to generate GeneScan files according to the manufacturer’s instructions.

The GeneScan files were analyzed using GeneMapper 4.1 software (Applied Biosystems, Foster City, CA, USA) to reveal capillary electrophoregrams of PCR amplified SSR-DNA fragments. Each specific peak (band) of SSR fingerprinting was marked as “1” or “A” when present and “0” or “C” when absent in each of the 40 strains of *X. albilineans*. The polymorphism information content (PIC) of loci was calculated according to the formula, where *P_ij_* is the frequency of *j*_th_ allele for *i*_th_ locus and summation extends over *n* alleles. A distance tree was constructed using clustering with the Unweighted Pair Group Method with Arithmetic Mean (UPGMA) in the SHAN program of NTSYS-PC 2.10e software (University of Kansas, Lawrence, USA).

### 2.5 Plant growth and inoculation with *X. albilineans*

Sugarcane plants with 1–2 fully expanded leaves (8–10 cm tall) of variety GT58 susceptible to leaf scald were used for inoculation with *X. albilineans*. To compare pathogenicity of the 40 *X. albilineans* strains, bacterial suspensions adjusted to 10^8^ CFU/mL were used for inoculation by the decapitation method. Briefly, the sugarcane stalk was cut 3–4 cm below the top visible dewlap leaf with scissors previously dipped in the bacterial inoculum. A sterile cotton ball was twined on the cut section before addition of 200 µl of bacterial inoculum (**Figure S1**). One day post inoculation (dpi), the cotton ball was removed from the cut section.

The 40 strains of *X. albilineans* were each inoculated to 35 sugarcane stalks and 35 control stalks were inoculated with only XAL medium (XAS liquid medium; Davis et al., 1994). Inoculated plants were randomly distributed and grown in an intelligent climate incubator (PLT-RGS-15PFC, Ningbo, China) at 28 °C, 65% humidity, and with a 16/8 h light/dark period. Three independent experiments were performed, which resulted in 105 inoculated plants per strain of the pathogen.

Furthermore, strains XaCN51 (high pathogenicity) and XaCN24 (low pathogenicity) of *X. albilineans* were used to investigate differential responses in sugarcane after inoculation with the leaf cutting method (Lin et al., 2018). The number of stalks inoculated per strain and the growing conditions of inoculated plants were identical to those described above. At each analysis time point [0, 12, and 24 hours post inoculation (hpi)], four leaf fragments were collected from each of 35 inoculated plants and these fragments were equally distributed into four pooled samples. These pooled samples with 35 leaf fragments each were used for the molecular and biochemical assays described below. The pooled sample for determination of bacterial population densities and the one for determination of gene expression were each divided in three biological subsamples. The pooled sample for measuring ROS and SA contents and the one for measuring oxidative enzyme activities were each divided in five biological subsamples.

### 2.6 Disease severity (DS) assessment

DS of leaf scald was assessed as reported by Rott et al. (1997) and Fu et al. (2021). Briefly, all inoculated stalks were rated individually at 7, 14, 21, 28 dpi, using a symptom severity scale ranging from 0 to 5 (**Table 1**). DS was calculated following the following formula: DS = [(0 × AP + 1 × FL + 2 × ML + 3 × CB + 4 × LN + 5 × PD)/5 × T] × 100, where AP, FL, ML, CB, LN, and PD are the number of stalks for each disease score and T is the total number of stalks. Mean DS at 28 dpi of each bacterial strain (three replications of 35 stalks) was used to perform a clustering analysis with Euclidean Distance implemented in SPSS software (version 18.0).

**Table 1:** Rating of leaf scald symptoms on a sugarcane stalk.

Score	Code	Symptoms
0	AP	None (Asymptomatic plant)
1	FL	One or two white pencil lines on the foliage (Few lines)
2	ML	More than two white pencil lines on the foliage (Many lines)
3	CB	Leaf chlorosis, bleaching, or yellowing
4	LN	Leaf necrosis
5	PD	Stalk/Plant death

### 2.7 Determination of bacterial population densities

Total DNA was extracted from leaf tissues with the CTAB reagent and the qPCR assay (TaqMan probe, *abc* gene, **Table S2**) was performed as described by Shi et al. (2021). Population densities of *X. albilineans* were determined in three leaf subsamples collected for each strain at 0, 12, and 24 hpi. Three technical replications were performed for each subsample.

### 2.8 Physico-biochemical assays

The activity of ROS, endogenous SA, and four antioxidant enzymes was determined with commercial kits and according to the manufacturers’ instructions (Beijing Solarbio Science & Technology Co., Ltd. China). The antioxidant enzymes were ascorbate peroxidase (APX, EC.1.11.1.11), superoxide dismutase (SOD, EC1.15.1.1), peroxidase (POD, EC1.11.1.7), and catalase (CAT, EC 1.11.1.6). Each leaf subsample (0.1 g each) was ground in 1 mL of 10 mM PBS buffer (pH = 7.4) using a freeze grinder (JXFSTPRP-CL, Shanghai, China). Ground leaf tissue was centrifuged at 2500 rpm for 20 min at 25 °C and the supernatant was used for subsequent determination of SA and ROS contents. Leaf fragments (0.1 g) of another subsample were ground in 1 mL extracting solution provided in the kit using the freeze grinder and, after homogenization, the tubes were centrifuged at 10,000 rpm for 10 min at 4 °C. The supernatant was used for determination of APX, SOD, POD, and CAT activities. ROS and SA contents were determined by sandwich ELISA and optical density was measured at 450 nm using a spectrophotometer, as per instructions of the corresponding kits (Beijing Solarbio Science & Technology Co., Ltd. China). ROS and SA concentrations were determined by comparison of OD values of each subsample with the standard curves. Following instructions of the extraction kits (Beijing Solarbio Science & Technology Co., Ltd. China), a spectrophotometric method was used to determine the activity of the four antioxidant enzymes at different visible wavelength, i.e., 240 nm (CAT), 290 nm (APX), 470 nm (POD), and 560 nm (SOD). The relative content or activity of each molecule was expressed as the ratio of values at 12 or 24 hpi versus values at 0 hpi. Five leaf subsamples were analyzed at each time point for each strain of the pathogen, and three technical replicates were performed per subsample.

### 2.9 Gene expression determined by qRT-PCR assay

Transcript expression of five genes was measured using a qRT-PCR assay with specific primer pairs (**Table S2**). These genes included the respiratory burst oxidase homolog gene (*ScRboh*) coding for the key enzyme in ROS production, and four SA signaling pathway related genes: *ScNPR3* (nonexpresser of pathogenesis-related gene 3), *ScTGA4* (encoding a TGA transcription factor), and *ScPR1* and *ScPR5* coding respectively for pathogenesis-related proteins PR1 and PR5. Total RNA was extracted from leaf subsamples and cDNA was synthesized by reverse transcription as previously described (Chu et al., 2020). The SYBR green dye method was used for qRT-PCR amplification with the QuantStudio 3 fluorescence quantitative PCR system (Applied Biosystems, USA). The qPCR mix contained 10.0 µL 2× ChamQ Universal SYBR qPCR Master Mix, 0.4 µL of each primer (10 µmol/µL), 1.0 µL cDNA (100 ng/µL), and 9.2 µL sterile high purity water. The amplification program was as follows: denaturation at 95 °C for 30 s, followed by 40 cycles at 95 °C for 10 s, and 60 °C for 30 s. The glyceraldehyde 3-phosphate dehydrogenase (*ScGAPDH*) gene was used as a reference gene, and the 2^-△△CT^ method was implemented to calculate relative gene expression. Three leaf subsamples were assayed at each time point for each strain of the pathogen, and three technical replicates were performed per subsample.

### 2.10 Statistical analysis

Data sets were compared by variance analysis (ANOVA) and Duncan’s test was used to identify mean differences at *p* < 0.05. All analyses were conducted using SPSS version 18.0 software (IBM, China).

## 3 Results

### 3.1 Molecular genotyping of worldwide strains of *X. albilineans* by MLSA

**Figure 1:**
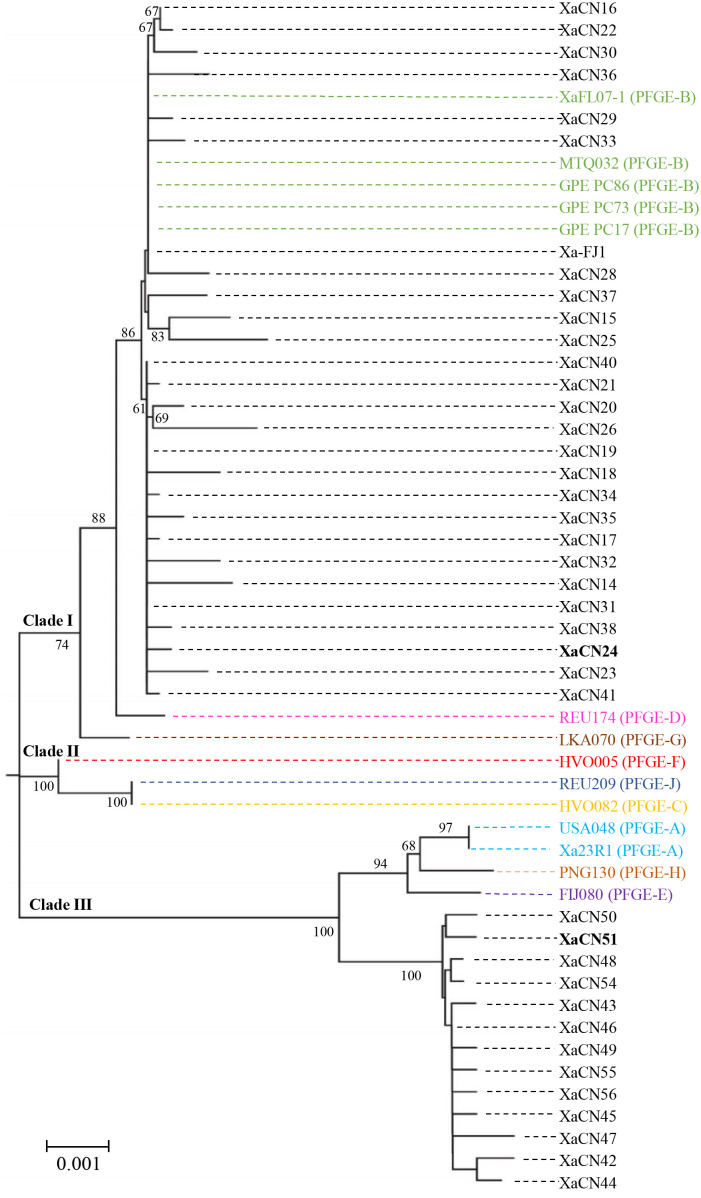
Neighbor-joining phylogenetic tree constructed with the concatenated sequence (4,165 nucleotides) of four housekeeping genes of 54 strains of *Xanthomonas albilineans* (40 from China and 14 from other geographical locations). Bootstrap values were determined for 1,000 replications and only bootstrap values >60% are shown at nodes. Strains XaCN24 and XaCN51 used for molecular investigations *in planta* are in bold. Strains from different pulsed-field gel electrophoresis (PFGE) groups (A-H, and -J) are written with different colors.

In a phylogenetic tree constructed with the concatenated sequences of four housekeeping genes, 54 strains of *X. albilineans* (this study = 40, and NCBI library = 14) were distributed in three major phylogenetic clades (**Figure 1**). Clade I contained a first sub-clade formed by 27 strains (including Xa-FJ1) from different provinces in China, one strain from USA/Florida (XAFL07-1), MTQ032 from Martinique, and three strains from Guadeloupe (GPE PC86, GPE PC73, and GPE PC17). The five strains from the USA and the Caribbean islands all belonged to PFGE group B. A second sub-clade of clade I was formed by a single strain (REU174) of PFGE group D. The last sub-clade of clade I contained only strain LKA070 representing PFGE group G. Clade II included three strains representing PFGE groups C (HVO082), F (HVO005), and J (REU209), and these strains were located on two different branches supported by a 100% bootstrap value.

Clade III was formed by two sub-groups supported by a 100% bootstrap value (**Figure 1**). The first one included two strains from Florida (USA048 and Xa23R1) belonging to PFGE group A, one strain from Papua New Guinea (PNG130) representing PFGE group H, and one from Fiji (FIJ080) belonging to PFGE group E. The second sub-clade of clade II contained 13 strains that were all from the city of Ruian in the Zhejiang province of China. This group of strains was named the Xa-RA group as it was not associated with any strain representing a PFGE group of *X. albilineans*.

### 3.2 Genetic diversity analysis among strains of *X. albilineans* from China using SSR markers

**Figure 2:**
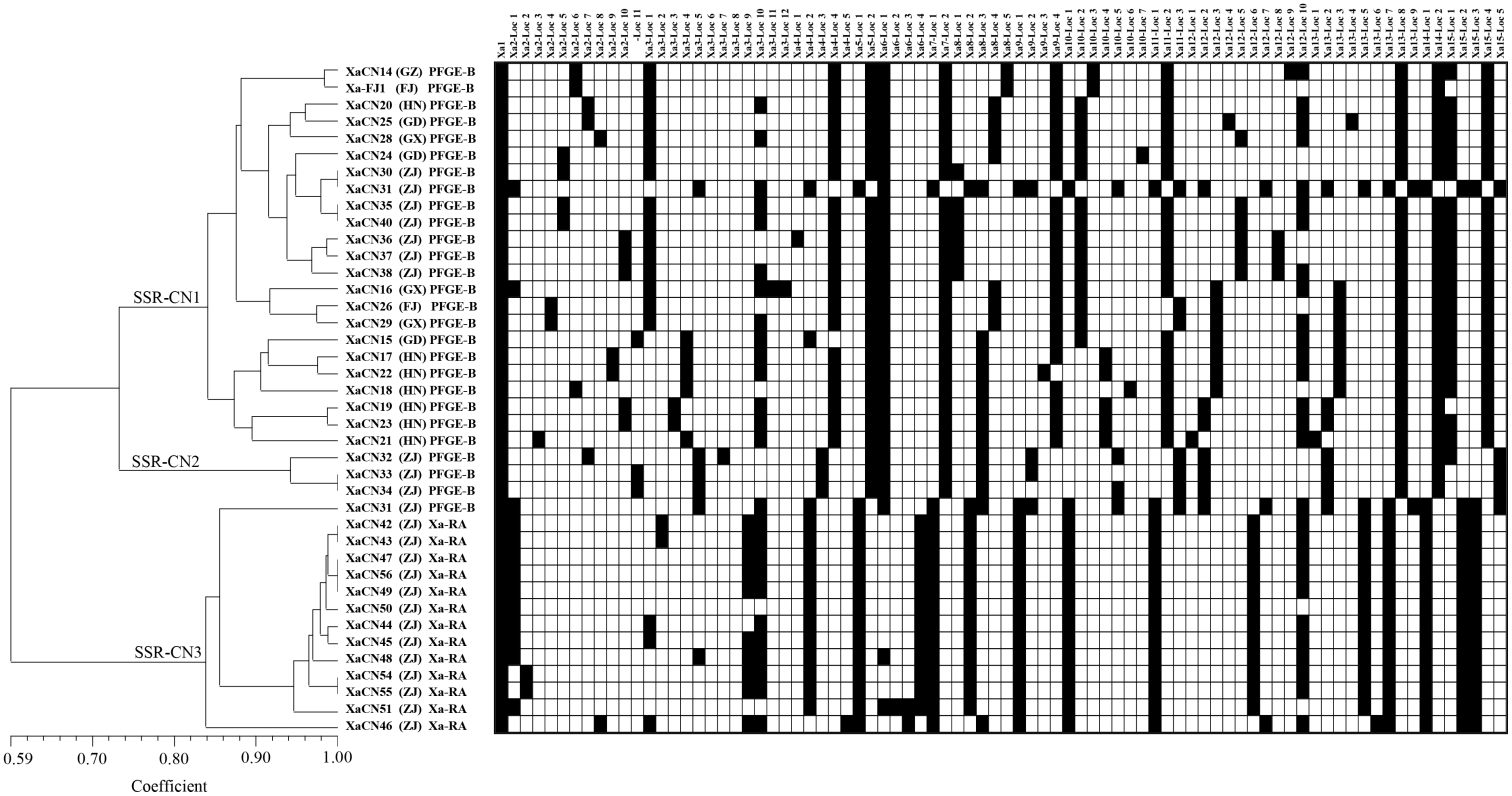
UPGMA phylogenetic tree of 40 strains of *Xanthomonas albilineans* from China based on 15 SSR marker loci (model = Maximum Composite Likelihood).

Fifteen primer pairs of SSR markers each amplified 1-12 loci for a total of 81 amplified loci present in the genome of the 40 strains of *X. albilineans* from China (**Table S3**). Among the 15 markers, 10 were highly polymorphic as their PIC value was > 0.50. Four markers were moderately polymorphic (0.25 ≤ PIC ≤ 0.50) and one showed no polymorphism (PIC = 0). The 40 strains of *X. albilineans* were distributed in three clades of an UPGMA phylogenetic tree, namely SSR-CN1, SSR-CN2, and SSR-CN3 (**Figure 2**). Clade SSR-CN1 included 23 strains from six provinces (Fujian, Guangdong, Guangxi, Guizhou, Hainan, and Zhejiang). All these strains were associated to strains of *X. albilineans* from other countries belonging to PFGE group B (**Figure 1** and section 3.1). Clade SSR-CN2 was formed by three strains (XaCN32, XaCN33, and XaCN34) from Wenling city in the Zhejiang province. These strains were also associated with foreign strains of PFGE group B based on MLSA. Clade SSR-CN3 consisted of 14 strains from the Zhejiang province. One of these 14 strains (XaCN31 from Wenling city) was associated to foreign strains of PFGE group B whereas the remaining 13 strains (from Ruian city) belonged to the Xa-RA group previously identified by MLSA.

### 3.3 Pathogenicity variation among strains of *X. albilineans*

The 40 strains of *X. albilineans* from China were inoculated into sugarcane variety GT58 and DS regularly increased from 7-28 dpi (**Figure S2**). At 28 dpi, mean DS varied between 30.2% (strain XaCN24) and 78.0% (strain XaCN51), revealing significant differences in pathogenicity among the strains of *X. albilineans* from China. The 40 stains of the pathogen were also distributed into three pathogenicity groups after a clustering analysis based on DS at 28 dpi (**Figure S3**). DS of the strains in the low pathogenicity group varied from 30.2-49.6%, from 47.3-64.2% in the medium group, and from 55.0-78.0% in the high pathogenicity group (**Table 2**).

**Table 2:** Distribution of 40 strains of *Xanthomonas albilineans* in different pathogenicity groups based on disease severity of sugarcane stalks 28 days post inoculation.

Pathogenicity group	Strain (Putative PFGE or other group)^a^	Number of strains	Disease severity (%)^b^	Mean ± SD^c^
Low	XaCN56 (Xa-RA), XaCN43 (Xa-RA), XaCN31 (PFGE-B), XaCN26 (PFGE-B), **XaCN24 (PFGE-B)**, XaCN20 (PFGE-B), XaCN16 (PFGE-B), XaCN14 (PFGE-B)	8	30.2–49.6	42.4 ± 5.8 a
Medium	Xa-FJ1 (PFGE-B), XaCN50 (Xa-RA), XaCN49 (Xa-RA), XaCN48 (PFGE-A), XaCN47 (PFGE-A), XaCN44 (Xa-RA), XaCN40 (PFGE-B), XaCN33 (PFGE-B), XaCN25 (PFGE-B), XaCN17 (PFGE-B), XaCN42 (Xa-RA), XaCN38 (PFGE-B)	12	47.3–64.2	55.0 ± 4.5 b
High	**XaCN51 (Xa-RA)**, XaCN30 (PFGE-B), XaCN23 (PFGE-B), XaCN22 (PFGE-B), XaCN15 (PFGE-B), XaCN36 (PFGE-B), XaCN54 (Xa-RA), XaCN46 (Xa-RA), XaCN55 (Xa-RA), XaCN19 (PFGE-B), XaCN45 (Xa-RA), XaCN35 (PFGE-B), XaCN32 (PFGE-B), XaCN21 (PFGE-B), XaCN34 (PFGE-B), XaCN37 (PFGE-B), XaCN18(PFGE-B), XaCN28 (PFGE-B), XaCN29 (PFGE-B), XaCN41 (PFGE-B)	20	55.0–78.0	66.3 ± 5.6 c

a Putative pulse-field gel electrophoresis (PFGE) group or other groups as determined in **Figure 1**. Strains in bold were used in this study for molecular investigations *in planta*.

b Minimum and maximum mean disease severity within a pathogenicity group.

c Mean and standard deviation (SD) were calculated with 105 plants inoculated per strain of *X. albilineans*. Means followed by the same letter are not significantly different at *P* = 0.05 according to Duncan’s test.

### 3.4 Bacterial population densities in sugarcane after inoculation with *X. albilineans*

**Figure 3:**
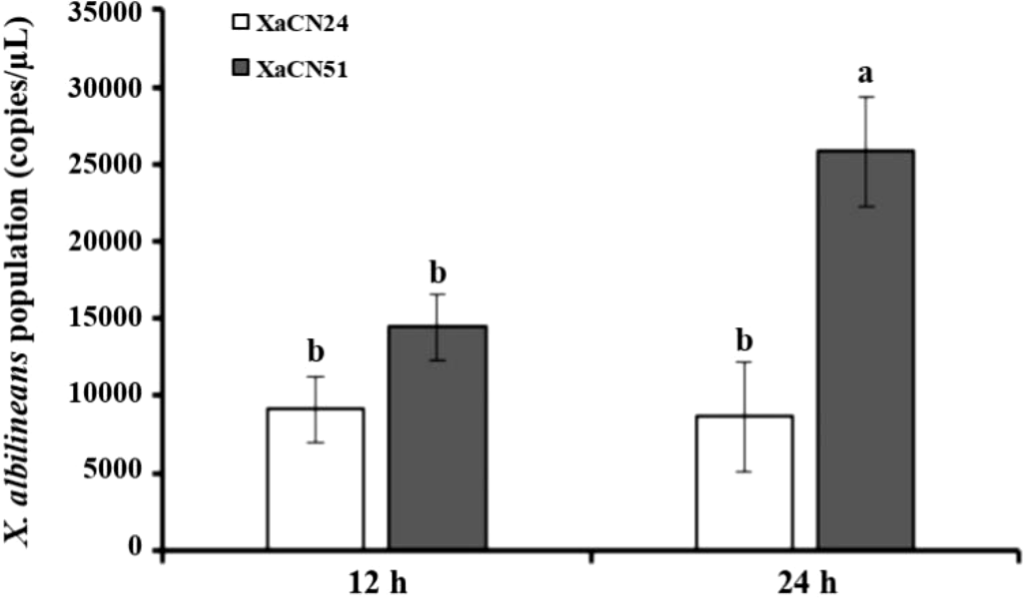
Population densities of two strains of *Xanthomonas albilineans* (XaCN24 and XaCN51) expressed as the number of genome copies determined by qPCR. Each vertical bar represents the mean ± standard deviation of three leaf subsamples collected 12 and 24 hours after inoculation and three technical replicates per subsample. Each leaf subsample is issued from a pooled sample of fragments collected from 35 inoculated leaves. Means with the same letter are not significantly different at *P* = 0.05 according to Duncan’s test.

Two strains of *X. albilineans* belonging to different evolutionary groups and differing in pathogenicity were used to investigate variation in population densities of the pathogen and sugarcane response mechanisms: strain XaCN24 (associated to group PFGE-B) with low pathogenicity and strain XaCN51 (Xa-RA group) with high pathogenicity. As determined by qPCR, mean bacterial population densities of the two strains were not significantly different in the leaves at 12 hpi (**Figure 3**). Leaf bacterial densities of XaCN51 increased from 1.45 x 10^4^ genome copies (GC)/μL at 12 hpi to 2.59 x 10^4^ GC/μL at 24 hpi. Population densities of XaCN24 were 9.12 × 10^3^ and 8.63 × 10^3^ GC/μL at 12 and 24 hpi, respectively. At 24 hpi, population densities of XaCN51 were significantly 3-fold higher than those of XaCN24.

### 3.5 Antioxidant capacity in sugarcane inoculated with *X. albilineans*

**Figure 4:**
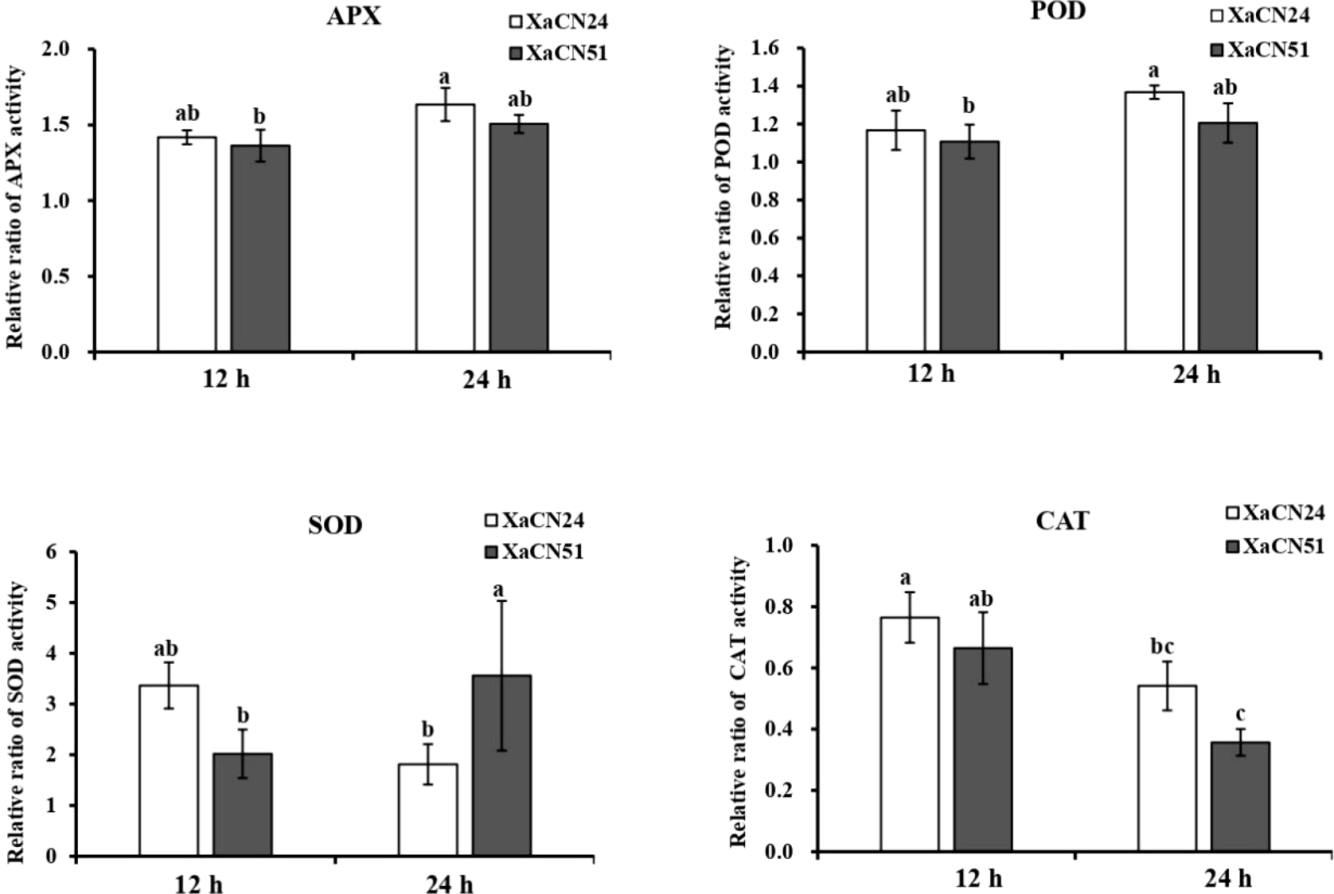
Changes of relative ratio of antioxidant enzyme activity in sugarcane leaves inoculated with two strains of *Xanthomonas albilineans* (XaCN24 and XaCN51). APX, ascorbate peroxidase; POD, peroxidase; SOD, peroxidase, and CAT, catalase. Each vertical bar represents the mean ± standard deviation of five leaf subsamples collected 12 and 24 hours after inoculation and three technical replicates per subsample. Each leaf subsample is issued from a pooled sample of fragments collected from 35 inoculated leaves. Means with the same letter are not significantly different at *P* = 0.05 according to Duncan’s test.

The dynamic changes of antioxidant capacity of enzymes APX, POD, SOD, and CAT were measured in sugarcane plants inoculated with strains XaCN24 and XaCN51. At 12 and 24h hpi, the relative activities of APX and POD were similar for both strains of the pathogen (**Figure 4**). For each enzyme and each bacterial strain, no significant increase or decrease of activity was observed from 12 to 24 hpi either. The relative activity of SOD in sugarcane inoculated with XaCN51 significantly increased 2.5 times from 12 to 24 hpi. In contrast, the relative activity of SOD in sugarcane inoculated with XaCN24 was not significantly different between 12 and 24 hpi (although a trend towards activity reduction was observed between the two time points). Relative activity of SOD was also higher as compared to APX and POD, regardless of inoculated strain and time point. The relative activity of CAT in sugarcane inoculated with each of the two strains was reduced from 12 to 24 hpi but was not significantly different between the two strains at each time point.

### 3.6 ROS content and *ScRboh* gene expression in sugarcane after inoculation with *X. albilineans*

**Figure 5:**
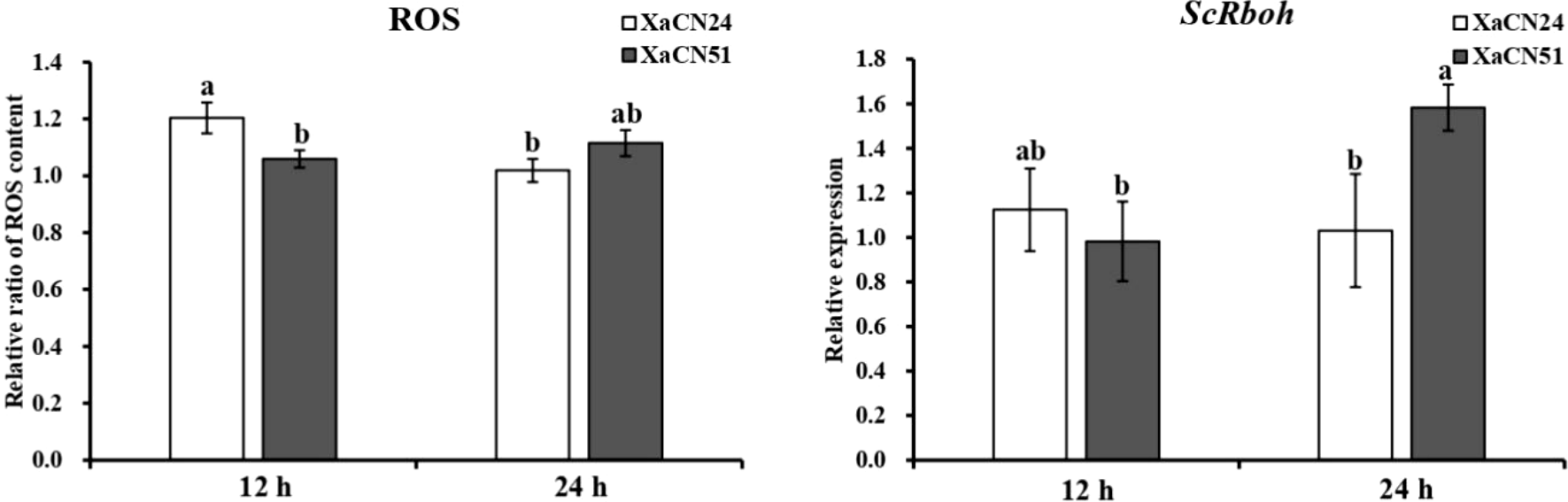
Changes of relative ratio of reactive oxygen species (ROS) content and relative expression of gene *ScRboh* gene in sugarcane leaves inoculated with two strains of *Xanthomonas albilineans* (XaCN24 and XaCN51). Each vertical bar represents the mean ± standard deviation of five (ROS) or three (*ScRboh*) leaf subsamples collected 12 and 24 hours after inoculation and three technical replicates per subsample. Each leaf subsample is issued from a pooled sample of fragments collected from 35 inoculated leaves. Means with the same letter are not significantly different at *P* = 0.05 according to Duncan’s test.

The relative level of ROS production was 18% lower in sugarcane leaves from 12 to 24 hpi with strain XaCN24 of *X. albilineans*. This level was not different between the two time points after inoculation with strain XaCN51 (**Figure 5**). The transcript level of gene *ScRboh*, which acts as a key enzyme in ROS production, was not significantly different between 12 and 24 hpi with strain XaCN24 of *X. albilineans* (**Figure 5**). After infection with XaCN51, the transcript level of *ScRboh* in sugarcane leaves increased by 60% from 12 to 24 hpi. At 24 hpi, expression level of *ScRboh* was also higher in leaves infected with XaCN51 than in leaves infected with XaCN24.

### 3.7 Endogenous SA level and SA-mediated gene expression in sugarcane infected by *X. albilineans*

**Figure 6:**
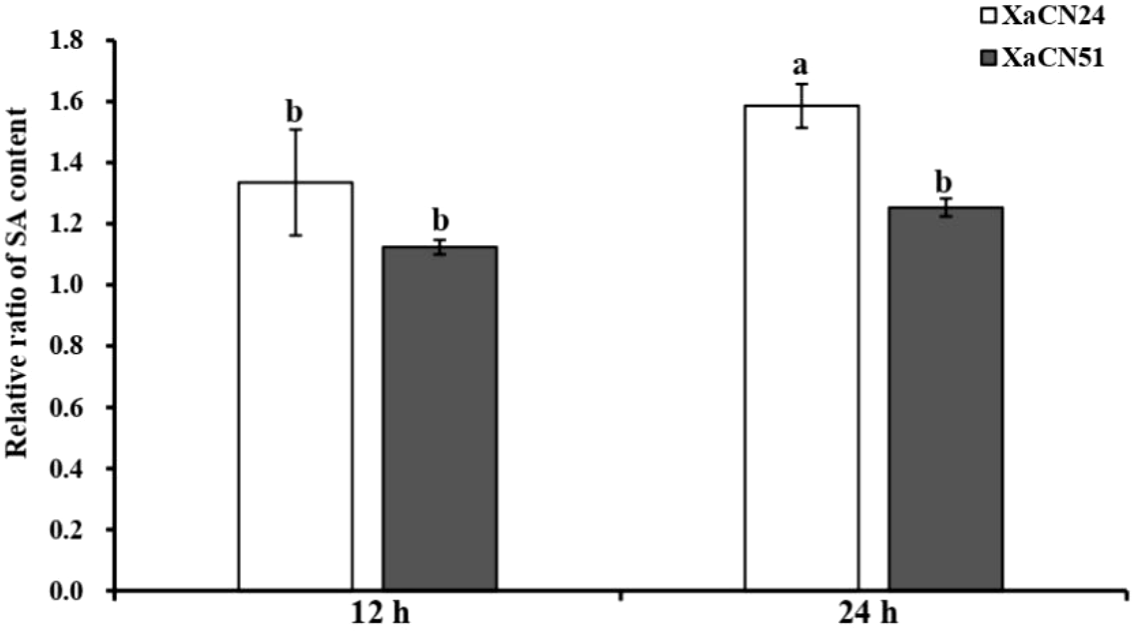
Changes of relative ratio of salicylic acid (SA) content in sugarcane leaves inoculated with two strains of *Xanthomonas albilineans* (XaCN24 and XaCN51). Each vertical bar represents the mean ± standard deviation of five leaf subsamples collected 12 and 24 hours after inoculation and three technical replicates per subsample. Each leaf subsample is issued from a pooled sample of fragments collected from 35 inoculated leaves. Means with the same letter are not significantly different at *P* = 0.05 according to Duncan’s test.

The relative SA content in leaves inoculated with XaCN51 was not different at 12 and 24 hpi (**Figure 6**). In contrast, this content increased from 12 to 24 hpi by 26% in leaves inoculated with XaCN24. At 24 hpi, the relative SA content was also higher in leaves infected with XaCN24 as compared to leaves infected with XaCN51.

**Figure 7:**
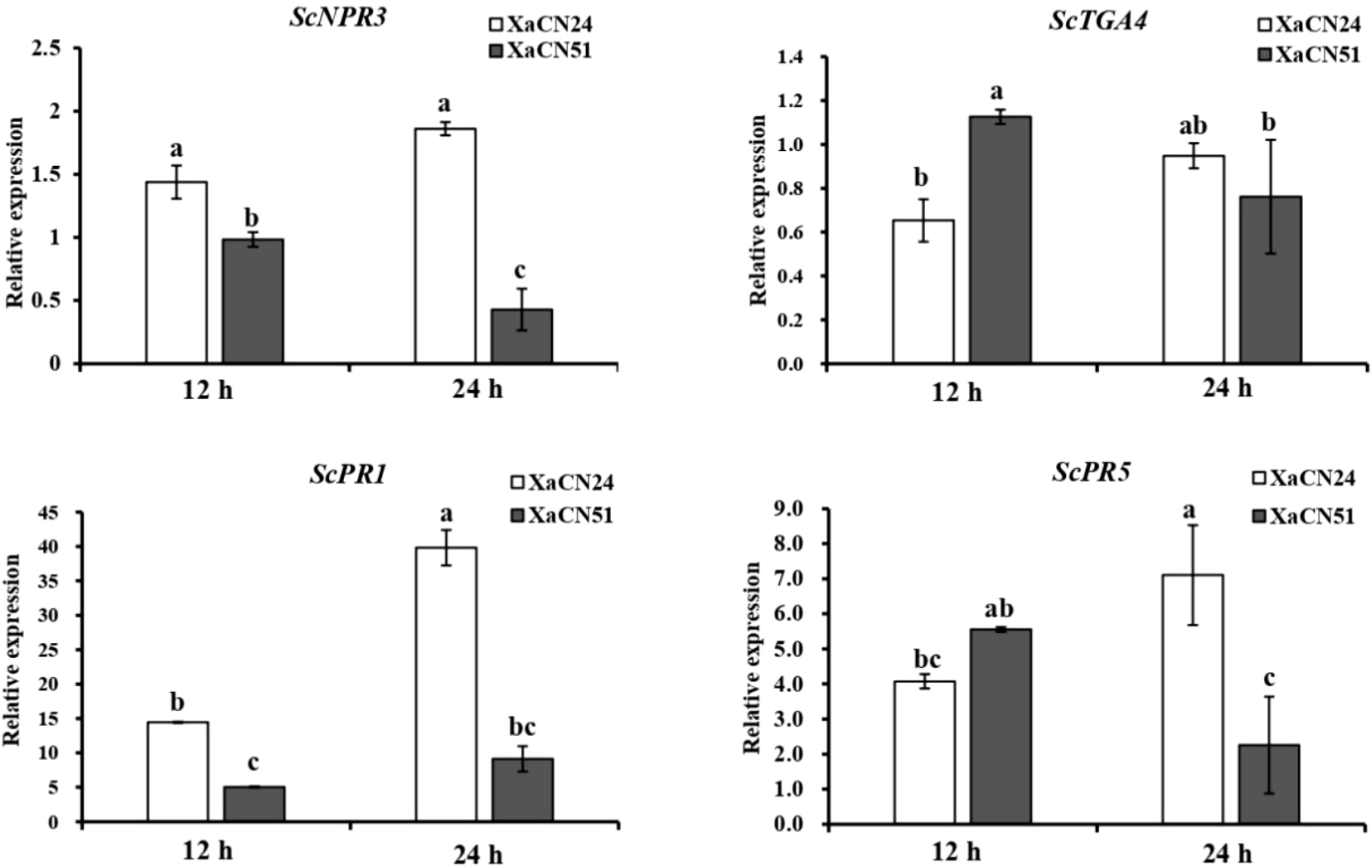
Expression pattern of salicylic acid (SA) signaling pathway related genes in sugarcane leaves inoculated with two strains of *Xanthomonas albilineans* (XaCN24 and XaCN51). Each vertical bar represents the mean ± standard deviation of three leaf subsamples collected 12 and 24 hours after inoculation and three technical replicates per subsample. Each leaf subsample is issued from a pooled sample of fragments collected from 35 inoculated leaves. Means with the same letter are not significantly different at *P* = 0.05 according to Duncan’s test.

In sugarcane leaves inoculated with *X. albilineans* XaCN24, the transcript level of SA-mediated genes *ScPR-1* and *ScPR-5* increased by 176 and 75% from 12 to 24 hpi, respectively (**Figure 7**). The transcript level of two additional SA-mediated genes (*ScNPR3* and *ScTGA4*) was not significantly different between the two time points, although a trend towards increase was observed.

After inoculation with *X. albilineans* XaCN51, the transcript level of genes *ScNPR-3*, *ScTGA4*, and *ScPR-5* decreased in sugarcane leaves by 50, 33, and 60% from 12 to 24 hpi, respectively (**Figure 7**). The transcript level of *ScPR1* was not significantly different between the two time points. At 24 hpi, the expression level of *ScNPR-3, ScPR-1*, and *ScPR5* was 280, 300, and 220% higher in leaves infected with XaCN24 than in leaves infected by XaCN51.

## 4 Discussion

Leaf scald occurs in most sugarcane-producing countries including China, which poses a threat to the worldwide sugarcane industry (Ricaud & Ryan, 1989; Rott & Davis, 2000; Meng et al., 2019). The genetic diversity of *X. albilineans* has been investigated using strains from various geographical locations (Alvarez et al., 1996; Davis et al., 1997; Champoiseau et al., 2006a; Meng et al., 2019; Cervantes-Romero et al., 2021). In China, sugarcane is mainly grown in the south, the east, and the west-south parts of the country where the ecological environments and climates are differing, thus contributing to the diversity of plant pathogenic bacteria. Up to now, only *X. albilineans* strains clustering with foreign strains of PFGE group B were reported in China (Zhang et al., 2017; Ntambo et al., 2019b). Nevertheless, recent observations suggested that other phylogenetic groups of *X. albilineans* occur in China (Duan et al., 2021).

In this study, presence in China of previously unreported strains belonging to a putatively new phylogenetic group (Xa-RA) was identified using MLSA and SSR markers. This group of strains (XaCN42-XaCHN51 and XaCN54-56) was collected from a specific chewing cane (Taoshanguozhe) that is an ancient clone of *S. officinarum* planted in the city of Ruian (Zhejiang province). This specific association of Xa-RA strains to a single host may suggest specific evolutionary driving forces for this group of strains. Additional investigations are needed to determine if the Xa-RA group of strains is unique to China or if it is connected to other strains in the world. Our study included sequences of strains representing all PFGE groups with the exception of PFGE group I (Martinique) that is not available in GenBank.

Besides genetic diversity, variation in pathogenicity also occurs among strains of *X. albilineans* from different countries or within the same geographical location (Mohamed et al., 1996; Champoiseau et al., 2006b; Tardiani et al., 2014; Wu et al., 2022). Resistance of sugarcane to leaf scald is closely associated with limited colonization of the host plant by the pathogen (Rott et al., 1994a; Rott et al., 1997; Garces et al., 2014). Consequently, disease severity and pathogen populations are lower in plants infected by low pathogenic strains of *X. albilineans* than in plants infected by high pathogenic strains (Mohamed et al., 1996). In our study, strains of *X. albilineans* varied from low to highly pathogenic and this variation was observed within and among sugarcane-producing provinces, and even within growing areas. No correlation was observed between the phylogenetic grouping and variation in pathogenicity of these strains, which confirms observations previously reported for *X. albilineans* (Champoiseau et al., 2006b; Tardiani et al., 2014) but also for other bacterial pathogens such as *X. citri* pv. *malvacearum* causing angular leaf spot and leaf blight of cotton (Kumar et al., 2018) and *Acidovorax avenae* subsp. *avenae* causing red stripe of sugarcane (Bertani et al., 2021).

Species of the genus *Xanthomonas* usually possess a Hypersensitive response and pathogenicity (Hrp) type III secretion system (Hrp-T3SS) that plays a critical role in inhibition of host defenses (Rossier et al., 1999; White et al., 2009). This secretion system allows Gram-negative plant pathogenic bacteria to inject bacterial effector proteins into the cytoplasm of the plant cell and thus participate in adaptation of a pathogen to its host (Büttner and Bonas, 2010; Timilsina et al., 2020). The sugarcane leaf scald pathogen, *X. albilineans*, does not possess this Hrp-T3SS and needs to rely on other secretion systems to interact with the proteins of the sugarcane host (Pieretti et al., 2009 and Pieretti et al., 2012; Zhang et al., 2020). These specific molecular interactions remain to be investigated, including among strains of the pathogen differing in pathogenicity.

The ROS, especially RBOH-dependent ROS, is a critical and effective component of plant disease resistance (Vidhyasekaran, 2014; Chen and Yang, 2020; Mittler et al., 2022). Overexpression of *GbRboh5/18* in *Gossypium barbadense* enhanced plant resistance to *Verticillium dahliae* by accumulation of ROS (Chang et al., 2020). Similarly, transcript levels of most *MeRboh* genes from cassava were increased following infection with *X. axonopodis* pv. *manihotis* (Huang et al., 2021). Genes *MeRbohB* and *MeRbohF* overexpressed in *Arabidopsis* also enhanced plant resistance to *Pseudomonas syringae* pv. tomato DC3000, most likely *via* the H_2_O_2_ signal transduction pathway and ROS generation (Huang et al., 2021). In our study, ROS production did not significantly change between 12 and 24 hpi but expression of Sc*Rboh* increased between these two time points in sugarcane leaves infected with the most pathogenic strain of *X. albilineans* (Xa-CN51). This suggested that ROS was also involved in response to *X. albilineans.* Additional time points after sugarcane inoculation need to be studied for better characterization of this defense mechanism, especially in varieties resistant to leaf scald.

Various antioxidants (non-enzymatic metabolites) and ROS scavenging enzymes such as SOD, CAT, POD, and APX are involved in scavenging H_2_O_2_ in plant cells (Afzal et al., 2014; Li and Ma, 2021). In our study, no significant changes were observed for the relative activities of POD and APX while the SOD activity increased between 12 and 24 hpi in sugarcane leaves infected with *X. albilineans* Xa-CN51 (the high pathogenic strain) but not with CXaCN24 (the low pathogenic strain). CAT activity decreased in leaves infected with Xa-CN51 and XaCN24 between 12 and 24 hpi, thus suggesting that SOD and CAT enzymes play important roles in sugarcane defense to infection by *X. albilineans.* Antioxidants SOD and CAT are crucial factors to modulate ROS scavenging and increased antioxidant activity is usually considered as a marker of reduced production and accumulation of H_2_O_2_ (Hong et al., 2021).

SOD or superoxide dismutase is a primary enzyme in the defense system against oxidative stress. SOD catalyzes the dismutation of superoxide anion $\left({\text{O}}_{2}^{\text{·−}}\right)$ to hydrogen peroxidase (H_2_O_2_) and molecular oxygen (O_2_), while CAT catalyzes the dismutation of H_2_O_2_ into water (H_2_O) and O_2_ (Manna et al., 2019; Li and Ma, 2021). Antioxidant effects of SOD and CAT enzymes are directly linked during conversion of superoxide to H_2_O_2_ and H_2_O_2_ to H_2_O and O_2_ (Xu et al., 2013). Usually, increased production of SOD and CAT corresponds to augmented tolerance of plants against adverse oxidative stresses (Xu et al., 2013; Suman et al., 2021). Rice mutants of gene *osnramp1* (natural resistance‐associated macrophage proteins) had an increased SOD activity and H_2_O_2_ content but a decreased activity of CAT after infection by *X. oryzae* pv. *oryzicola*, *Magnaporthe oryzae*, and *Ustilaginoidea virens* (Chu et al., 2022a). This contributed to enhanced broad‐spectrum resistance of rice against bacterial and fungal pathogens.

A similar response was found herein for sugarcane in response to infection by *X. albilineans*, especially in sugarcane infected by highly pathogenic strain XaCN51. Sugarcane variety GT58 susceptible to leaf scald appeared to regulate ROS homoeostasis by upregulating *ScRboh* expression (contributing to ROS production) and enhancing H_2_O_2_ production (elevating SOD and decreasing CAT activities) during early host-pathogen interaction. Notably, excessive ROS production triggers an oxidative stress response, cellular damage, and cell death (Manna et al., 2019). Furthermore, excess of H_2_O_2_ causes chloroplast and peroxisome autophagy and programmed cell death (Smirnoff and Arnaud, 2019). Increased H_2_O_2_ altering ROS homoeostasis may result in increased susceptibility of sugarcane to *X. albilineans*. On the other hand, this pathogen (especially strain Xa-CN51) may be tolerant to high concentrations of H_2_O_2_. In barley, H_2_O_2_ levels were higher in inoculated leaves of a susceptible variety than in a more resistant one during later stages of Ramularia leaf spot. The causal agent of this disease, *Ramularia collo-cygni*, was also able to grow *in vitro* on media containing relatively high concentrations of H_2_O_2_ (McGrann and Brown, 2018).

Among phytohormones, SA is involved in the hypersensitive response (HR) and systemic acquired resistance (SAR) of plants. Consequently, SA plays a critical role in plant defense against biotrophic and semi-biotrophic pathogens (Yang et al., 2015; Peng et al., 2021). Accumulated SA activates SA-signaling pathways to induce the expression of defense-related genes such as PR1 and PR5 (Chu et al., 2022b). To achieve successful infection of a plant, pathogenic organisms have evolved three main strategies to disrupt SA-mediated defense genes: i/disruption of SA biosynthesis by targeting the isochorismate synthase I pathway, ii/reduction of SA accumulation by conversion of SA into its inactive derivatives, and iii/various mechanisms to interfere with SA downstream signaling (Qi et al., 2018). An effector produced by *Ralstonia solanacearum*, causing wilt of arabidopsis (*Arabidopsis thaliana*) and tomato (*Solanum lycopersicum*), disrupts SA signaling by inhibiting TGA activity to establish successful infections (Qi et al., 2022). In our study, the highly pathogenic strain of *X. albilineans* was associated with low levels of endogenous SA and reduced expression of genes involved in the SA pathway as compared to the low pathogenic strain. Similarly, expression of SA-mediated sugarcane defense genes *PR1* and *PR5* was reduced after infection with XaCN51 whereas expression of these two genes was upregulated in response to the low pathogenic strain of *X. albilineans.* This is evidence for involvement of these genes and SA in resistance of sugarcane to leaf scald.

The nonexpressor of pathogenesis-related gene 1 (NPR1) protein is an SA receptor of the plant that promotes SA-induced defense gene expression (Backer et al., 2019; Chen et al., 2021). However, when endogenous concentrations of SA are low, NPR3/4 (paralogs of NPR1) inhibit the expression of genes downstream of the SA signal transduction pathway (Ding and Ding, 2020; Peng et al., 2021). SA also regulates ROS homeostasis and the antioxidant defense system, both at physiological and molecular levels (Saleem et al., 2021). In *Nicotiana benthamiana*, both local and systemic resistances to tobacco mosaic virus were differentially modulated by SA and ROS under glutathione mediation (Zhu et al., 2021). Lower levels of SA in sugarcane infected by the highly pathogenic strain of *X. albilineans* may therefore also inhibit expression of sugarcane genes involved in resistance to leaf scald. Molecular cross talks between the SA and ROS signals during the sugarcane and *X. albilineans* interactions remain to be further investigated.

## 5 Conclusion

We demonstrated for the first time in this study that at least two phylogenetic groups of *X. albilineans* were present in sugarcane-producing regions of China. Forty strains of *X. albilineans* were distributed into three pathogenicity groups (i.e. low, medium, and high) after inoculation of a sugarcane variety susceptible to leaf scald. Based on qPCR data, population densities of highly pathogenic strain XaCN51 were higher in infected leaves than population densities of low pathogenic strain XaCN24. ROS production and the antioxidant defense system, as well as SA generation and SA-mediated genes (*ScNPR3*, *ScTGA4*, *ScPR1*, and *ScPR5*), participated in the response of sugarcane to infection by *X. albilineans.* Preformation and gene expression of these stress-related signal molecules differed according to the two strains of the pathogen varying in pathogenicity. This foundation work should be useful for further research on the defense mechanisms of sugarcane in response to an attack of *X. albilineans* and possibly other pathogens.

## Data availability statement

The original contributions presented in the study are publicly available. This data can be found in the text.

## Author contributions

Conceptualization, J-YZ and S-JG. Writing—original draft preparation, J-YZ and YS. Writing—review and editing, PR and S-JG. Data curation, JC and YS. Experiments preformation, JC, YS, H-YF, and M-TH. Supervision, funding acquisition, and project administration, S-JG. All authors contributed to the article and approved the submitted version.
