# Supplementary material for: Sugarcane responses to two strains of Xanthomonas albilineans differing in pathogenicity through a differential modulation of salicylic acid and reactive oxygen species
Source: Front Plant Sci. 2022 Dec 15;13:1087525. doi: 10.3389/fpls.2022.1087525 (PMC9798216; doi:10.3389/fpls.2022.1087525)
Supplement: Supplementary file 1 [file DataSheet_1.zip › Supplementary file-updated 20221201.docx]

**
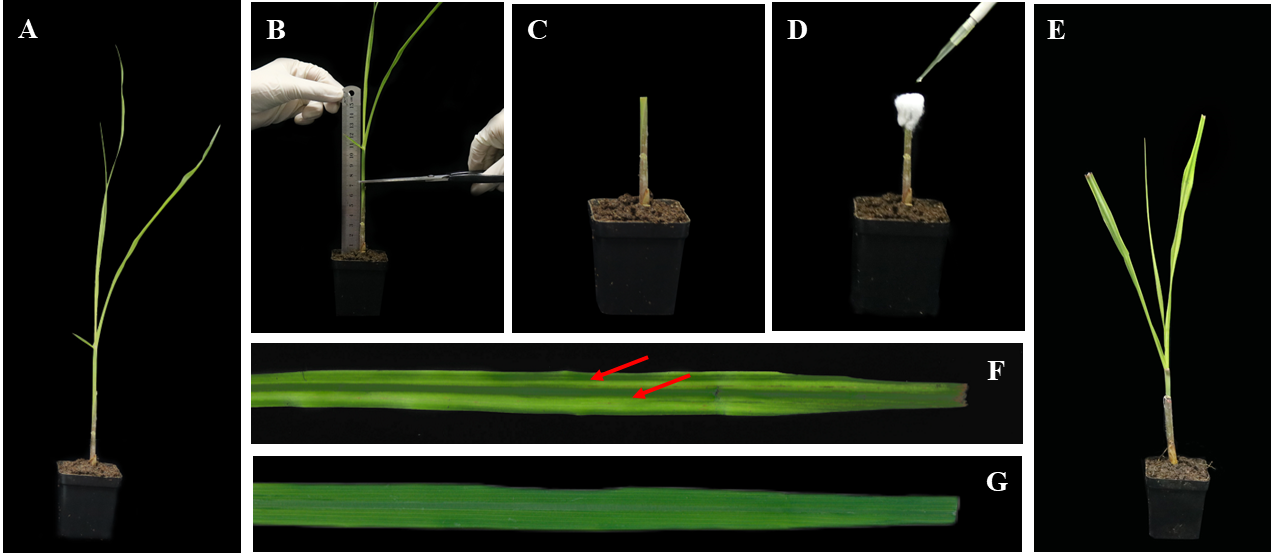
**

**Figure S1.** Inoculation of a young sugarcane plant with *Xanthomonas albilineans* using a decapitation method. A. Healthy sugarcane plant of variety GT58 before inoculation; B and C. The sugarcane shoot was cut with scissors below the top visible dewlap leaf; D. A cotton ball was twined on the cut section before addition of 200 µl of bacterial inoculum; E. Sugarcane shoot exhibiting newly developed leaves 14 days post inoculation (dpi); F. Typical pencil line symptoms with slight necrosis (red arrows) observed on the leaf blade 14 dpi; G. Symptomless leaf of a control plant 14 dpi with sterile water.


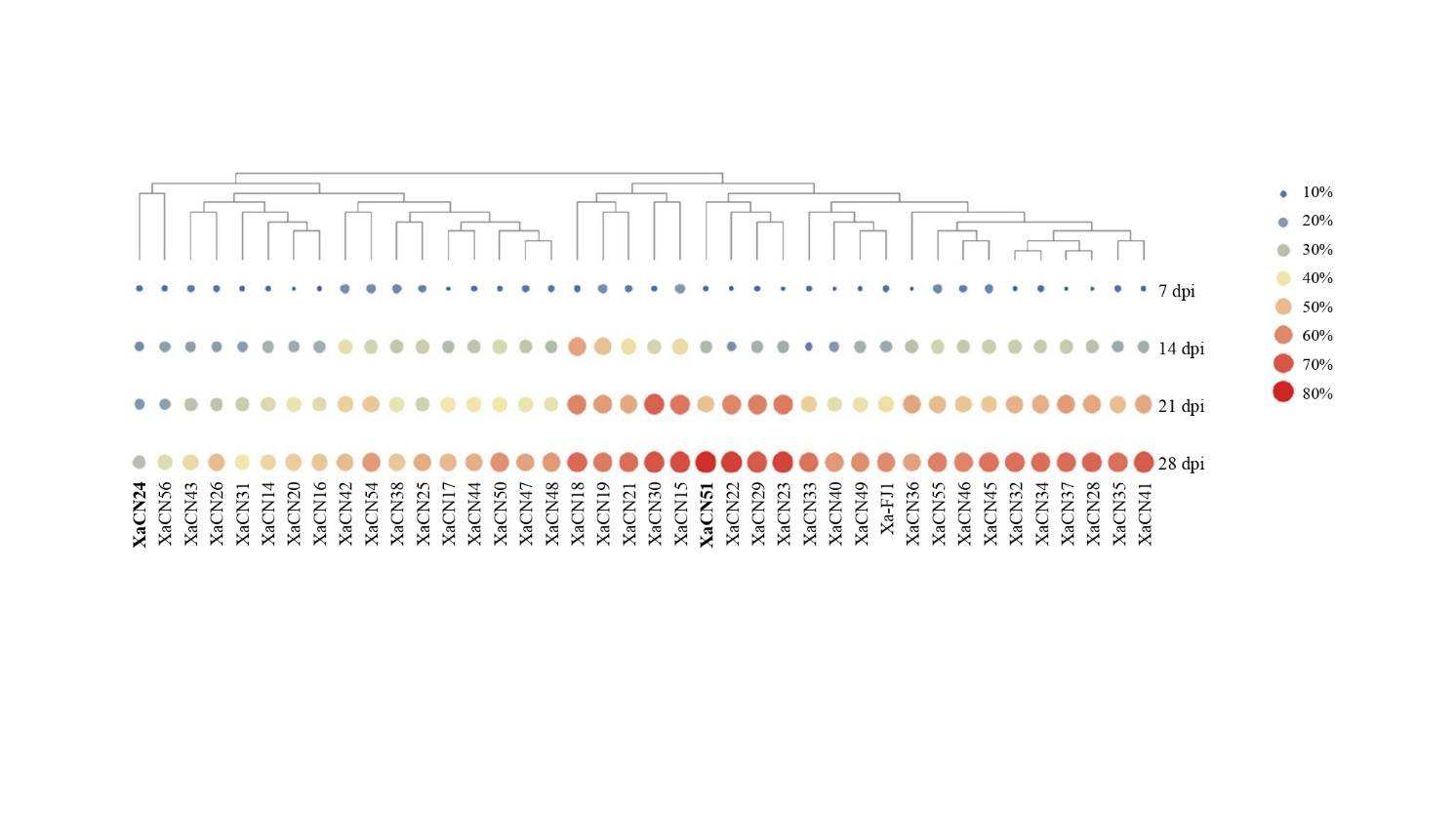


**Figure S2**. Heatmap of disease severity (DS) of sugarcane variety GT58 inoculated with 40 strains of *X. albilineans* from China. Data are from 7-28 days post inoculation. Colored circles correspond to mean disease severity at each time point and for each strain. Each circle is the mean DS of 105 plants (three independent experiments of 35 plants each). The two strains used for molecular studies are indicated in bold.


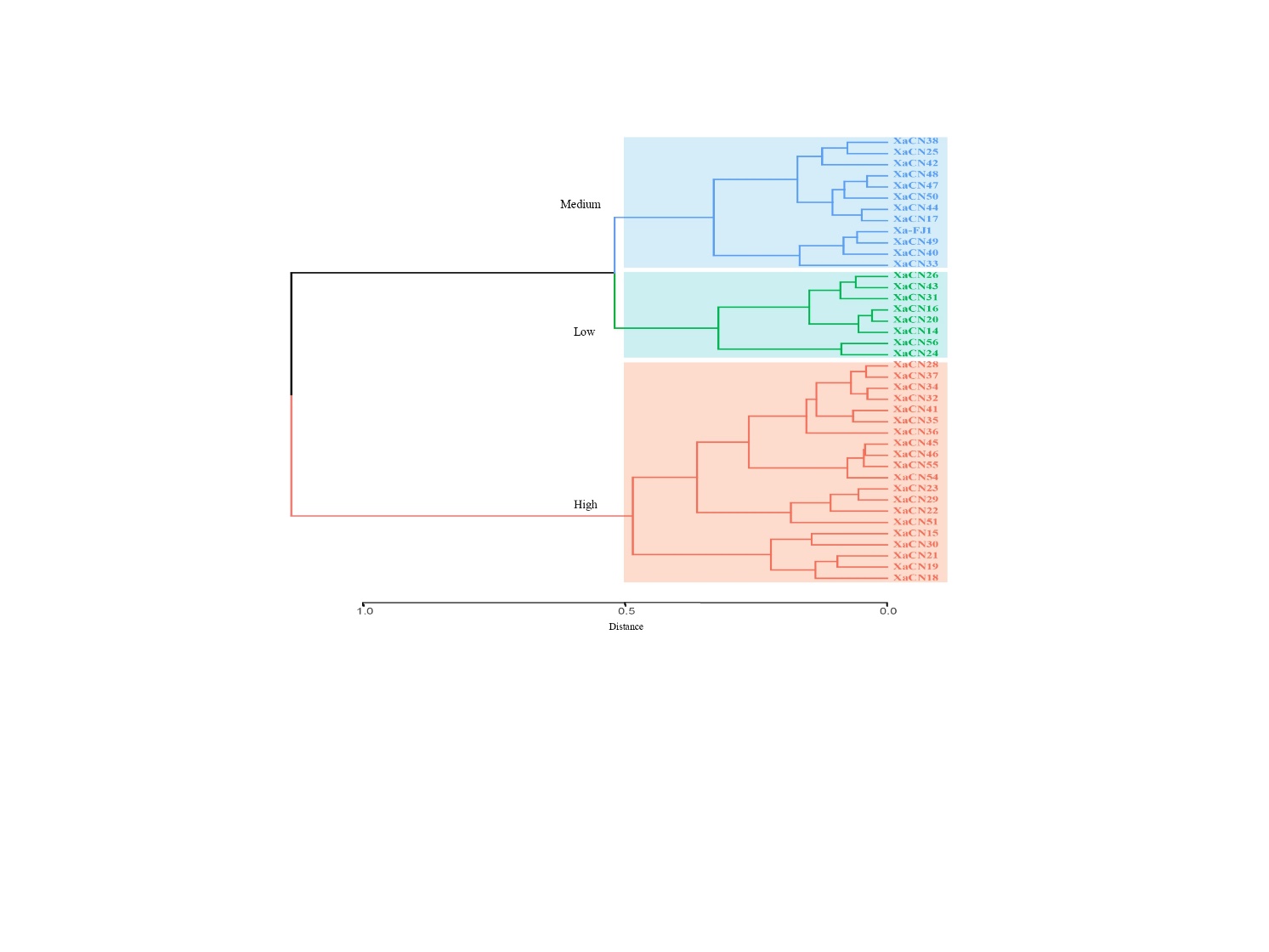


**Figure S3.** Cluster analysis based on disease severity of 40 strains of *X. albilineans*. Data are from sugarcane plants 28 days post inoculation.

**Table S1** Characteristics of 54 strains of *Xanthomonas albilineans* from China and other countries.

| No. | Strain | Host variety | Tissue | Sampling location | Sampling date |
| --- | --- | --- | --- | --- | --- |
| 1 | Xa-FJ1 | Yuegan 48 (*Saccharum* spp.) | leaf | China: Zhangzhou, Fujian | 2015/01 |
| 2 | XaCN14 | Qiantang 8 (*Saccharum* spp.) | leaf | China: Xingyi, Guizhou | 2018/08 |
| 3 | XaCN15 | Guitang 08-1589 (*Saccharum* spp.) | leaf | China: Zhanjiang, Guangdong | 2018/01 |
| 4 | XaCN16 | GUC37 (*Saccharum* spp.) | leaf | China: Fusui, Guangxi | 2018/01 |
| 5 | XaCN17 | ROC20 (*Saccharum* spp.) | leaf | China: Wuzhishan, Hainan | 2019/06 |
| 6 | XaCN18 | Yuetang 92-1287 (*Saccharum* spp.) | leaf | China: Wuzhishan, Hainan | 2019/06 |
| 7 | XaCN19 | Funong 93-23 (*Saccharum* spp.) | leaf | China: Wuzhishan, Hainan | 2019/06 |
| 8 | XaCN20 | ROC20 (*Saccharum* spp.) | leaf | China: Wuzhishan, Hainan | 2019/06 |
| 9 | XaCN21 | HoCP 93-750 (*Saccharum* spp.) | leaf | China: Sanya, Hainan | 2019/06 |
| 10 | XaCN22 | *Saccharum* spp. hybrid 16139 | leaf | China: Danzhou, Hainan | 2019/06 |
| 11 | XaCN23 | *Saccharum* spp. hybrid | leaf | China: Danzhou, Hainan | 2019/06 |
| 12 | XaCN24 | Yunzhe 11-1204 (*Saccharum* spp.) | leaf | China: Zhanjiang, Guangdong | 2019/06 |
| 13 | XaCN25 | Regan 15-285 (*Saccharum* spp.) | leaf | China: Zhanjiang, Guangdong | 2019/06 |
| 14 | XaCN26 | *Pennisetum purpureum* | leaf | China: Fuzhou, Fujian | 2019/04 |
| 15 | XaCN28 | *Saccharum* spp. hybrid | leaf | China: Wuming, Guangxi | 2019/05 |
| 16 | XaCN29 | Yuetang 93-159 (*Saccharum* spp.) | leaf | China: Fuzhou, Fujian | 2019/05 |
| 17 | XaCN30 | Guangdonghaungpi (*S. officinarum*) | leaf | China: Wenling, Zhejiang | 2019/06 |
| 18 | XaCN31 | Guangdonghaungpi (*S. officinarum*) | leaf | China: Wenling, Zhejiang | 2019/06 |
| 19 | XaCN32 | Guangdonghaungpi (*S. officinarum*) | leaf | China: Wenling, Zhejiang | 2019/06 |
| 20 | XaCN33 | Guangdonghaungpi (*S. officinarum*) | leaf | China: Wenling, Zhejiang | 2019/06 |
| 21 | XaCN34 | Guangdonghaungpi (*S. officinarum*) | leaf | China: Wenling, Zhejiang | 2019/06 |
| 22 | XaCN35 | Guangdonghaungpi (*S. officinarum*) | leaf | China: Wenling, Zhejiang | 2019/06 |
| 23 | XaCN36 | Guangdonghaungpi (*S. officinarum*) | stalk | China: Wenling, Zhejiang | 2019/06 |
| 24 | XaCN37 | Badila (*S. officinarum*) | leaf | China: Wenling, Zhejiang | 2019/06 |
| 25 | XaCN38 | Guangdonghaungpi (*S. officinarum*) | leaf | China: Wenling, Zhejiang | 2019/06 |
| 26 | XaCN40 | Guangdonghaungpi (*S. officinarum*) | leaf | China: Wenling, Zhejiang | 2019/06 |
| 27 | XaCN41 | Guangdonghaungpi (*S. officinarum*) | stalk | China: Wenling, Zhejiang | 2019/06 |
| 28 | XaCN42 | Taoshanguozhe (*S. officinarum*) | leaf | China: Ruian, Zhejiang | 2019/08 |
| 29 | XaCN43 | Taoshanguozhe (*S. officinarum*) | leaf | China: Ruian, Zhejiang | 2019/08 |
| 30 | XaCN44 | Taoshanguozhe (*S. officinarum*) | leaf | China: Ruian, Zhejiang | 2019/08 |
| 31 | XaCN45 | Taoshanguozhe (*S. officinarum*) | leaf | China: Ruian, Zhejiang | 2019/08 |
| 32 | XaCN46 | Taoshanguozhe (*S. officinarum*) | leaf | China: Ruian, Zhejiang | 2019/08 |
| 33 | XaCN47 | Taoshanguozhe (*S. officinarum*) | stalk | China: Ruian, Zhejiang | 2019/08 |
| 34 | XaCN48 | Taoshanguozhe (*S. officinarum*) | leaf | China: Ruian, Zhejiang | 2019/08 |
| 35 | XaCN49 | Taoshanguozhe (*S. officinarum*) | stalk | China: Ruian, Zhejiang | 2019/08 |
| 36 | XaCN50 | Taoshanguozhe (*S. officinarum*) | leaf | China: Ruian, Zhejiang | 2019/08 |
| 37 | XaCN51 | Taoshanguozhe (*S. officinarum*) | stalk | China: Ruian, Zhejiang | 2019/08 |
| 38 | XaCN54 | Taoshanguozhe (*S. officinarum*) | leaf | China: Ruian, Zhejiang | 2019/08 |
| 39 | XaCN55 | Taoshanguozhe (*S. officinarum*) | stalk | China: Ruian, Zhejiang | 2019/08 |
| 40 | XaCN56 | Taoshanguozhe (*S. officinarum*) | stalk | China: Ruian, Zhejiang | 2019/08 |
| 41 | FIJ080 |  |  | Fiji | 1961 |
| 42 | GPE PC73 |  |  | France: Guadeloupe | 2003 |
| 43 | GPE PC17 |  |  | France: Guadeloupe | 2003 |
| 44 | GPE PC86 |  |  | France: Guadeloupe | 2003 |
| 45 | HVO005 |  |  | Burkina Faso | 1980 |
| 46 | HVO082 |  |  | Burkina Faso | 1989 |
| 47 | LKA070 |  |  | Sri Lanka | 1962 |
| 48 | MTQ032 |  |  | France: Martinique | 1932 |
| 49 | PNG130 |  |  | Papua New Guinea | 1993 |
| 50 | REU174 |  |  | France: Reunion Island | 1995 |
| 51 | REU209 |  |  | France: Reunion Island | 1995 |
| 52 | USA048 |  |  | USA: Florida | 1986 |
| 53 | Xa23R1 |  |  | USA: Florida | 1996 |
| 54 | XaFL07-1 |  |  | USA: Florida | 2007 |

**Table S2** Primer pairs used to amplify, clone, and sequence four housekeeping genes, to determine bacterial population densities by qPCR, and to analyze transcript expression by RT-qPCR.

| Target Gene | Primer | Sequence (5’→3’) | Tm (℃) | Fragment size (bp) | Amplification method and conditions |
| --- | --- | --- | --- | --- | --- |
| *atpD* | atpD-F2 | GGGCAAGATCGTTCAGAT | 50 | 868 | PCR: 94 ℃, 2 min; 94 ℃, 1 min, 50 ℃, 90 s, 72 ℃, 90 s, 30 cycles; 72 ℃, 10 min |
|  | atpD-R2 | GTTCTTGGTGGAGGTGAT |  |  |  |
| *glnA* | glnA-F2 | GGTTAAGGACAACAAGATCG | 52 | 1095 | PCR: 94 ℃, 2 min; 94 ℃, 1 min, 52 ℃, 90 s, 72 ℃, 90 s, 30 cycles; 72 ℃,10 min |
|  | glnA-R2 | GCGGCRAAGGTCAGGTAG |  |  |  |
| *gyrB* | XgyrB1F | ACGAGTACAACCCGGACAA | 52 | 904 | PCR: 94 ℃, 2 min; 94 ℃, 1 min, 52 ℃, 90 s, 72 ℃, 90 s, 30 cycles; 72 ℃, 10 min |
|  | XgyrB1R2 | CCCATCAAGGTGCTGAAAAT |  |  |  |
| *rpoD* | rpoD-F1 | ATGGCCAACGAACGTCCTGC | 56 | 1298 | PCR: 94 ℃, 2 min; 94 ℃, 1 min, 56 ℃, 90s, 72 ℃, 90 s, 30 cycles; 72 ℃, 10 min |
|  | rpoDR3 | AACTTGTAGCCGCGACGGTATTC |  |  |  |
| *abc* | XaABCF3 | CGGCCAGAAGCAGAGAATCC | 57 | 123 | qPCR: 95 ℃, 2 min; 5 s at 95℃ 5s, 60℃, 30 s, 40 cycle |
|  | XaABCR3 | GCGATCTCGTTGTTGATGCG |  |  |  |
|  | XaABCP3 | TGCTCGCGAGAGCGCTCTACA |  |  |  |
| *ScRboh* | q-ScRboh-F | ATTCCTTCAGGCGGAGTGTG | 57 | 109 | RT-qPCR: 95 ℃, 30 s; 95 ℃, 10 s, 60 ℃, 30 s, 40 cycles; 95 ℃, 15 s, 60 ℃, 60 s, 95 ℃, 15 s |
|  | q-ScRboh-R | TAGGAATGCACCACCAGCAG |  |  |  |
| *ScNPR3* | q-ScNPR3-F | CCTATGGCGGTGGAAGATGC | 56 | 143 |  |
|  | q-ScNPR3-R | TCTGTGGTGTCTGCTTGTGC |  |  |  |
| *ScTGA4* | q-ScTGA02-1-F | AGCCCTAGAACAGAAACATCA | 58 | 106 |  |
|  | q-ScTGA02-1- R | CAACCACCTCGCATACTCC |  |  |  |
| *ScPR1* | q-ScPR1-F | CCTATGGCGGTGGAAGATGC | 55 | 110 |  |
|  | q-ScPR1-R | TCTGTGGTGTCTGCTTGTGC |  |  |  |
| *ScPR5* | q-ScPR5-F | GGCGGCAAAGATTTCTACGATG | 59 | 107 |  |
|  | q-ScPR5-R | GAGACACAGCCTGCGTATTTGC |  |  |  |
| *ScGAPDH* | GAPDH-F | CACGGCCACTGGAAGCA | 58 | 110 |  |
|  | GAPDH-R | TCCTCAGGGTTCCTGATGCC |  |  |  |

**Table S3** Polymorphism information content (PIC) of 15 SSR markers using capillary electrophoresis of 40 strains of *X. albilineans.*

| Marker  No. | Marker  name | Amplicon range in bp | Number of theoretical fragments | Number of amplified fragments | PIC | Tm (℃) |
| --- | --- | --- | --- | --- | --- | --- |
| 1 | IACXa1 | 97 | 1 | 1 | 0 | 64 |
| 2 | IACXa2 | 170-341 | 12 | 11 | 0.85 | 59 |
| 3 | IACXa3 | 124-524 | 8 | 12 | 0.88 | 64 |
| 4 | IACXa4 | 296-492 | 1 | 5 | 0.6 | 61 |
| 5 | IACXa5 | 371-476 | 2 | 2 | 0.46 | 59 |
| 6 | IACXa6 | 174-219 | 4 | 4 | 0.48 | 61 |
| 7 | IACXa7 | 135-155 | 3 | 2 | 0.46 | 64 |
| 8 | IACXa8 | 108-153 | 3 | 5 | 0.77 | 60 |
| 9 | IACXa9 | 120-148 | 1 | 4 | 0.59 | 59 |
| 10 | IACXa10 | 101-645 | 4 | 7 | 0.75 | 58 |
| 11 | IACXa11 | 108-132 | 4 | 2 | 0.6 | 59 |
| 12 | IACXa12 | 116-440 | 6 | 10 | 0.89 | 60 |
| 13 | IACXa13 | 122-340 | 5 | 9 | 0.77 | 59 |
| 14 | IACXa14 | 126-132 | 2 | 2 | 0.46 | 59 |
| 15 | IACXa15 | 115-146 | 2 | 5 | 0.61 | 60 |
